# Belladonna in Rigidity of the Os Tincæ, Os Eaternum and Perinæum, in Parturition

**Published:** 1834

**Authors:** 


### II. ANALYTICAL.

1. *Belladonna in Rigidity of the os tincæ, os externum and perinæum, in parturition.*—Our attention has been directed to this subject, by an anonymous writer, in the *Boston Medical and Surgical Journal*, who stands forth as the advocate of the extract of Belladonna in these rigidities. It is to be applied to the os uteri, and perinæum, and great relaxation is said speedily to follow. We are disposed to think, that in a majority of cases, copious blood letting, which we have almost always found successful, should precede the application of the extract, which, in general, will be then unnecessary.

